# The effect of lung recruitment maneuvers on post-operative pulmonary complications for patients undergoing general anesthesia: A meta-analysis

**DOI:** 10.1371/journal.pone.0217405

**Published:** 2019-05-29

**Authors:** Yu Cui, Rong Cao, Gen Li, Tianqing Gong, Yingyu Ou, Jing Huang

**Affiliations:** 1 Department of Anesthesiology, Chengdu Women’s and Children’s Central Hospital, Chengdu, Sichuan, China; 2 Department of Anesthesiology, Vanderbilt University Medical Center, Nashville, Tennessee, United States of America; Heidelberg University Hospital, GERMANY

## Abstract

**Background:**

Respiratory function would be impaired during general anesthesia period. Researchers devoted their energies to finding effective strategies for protecting respiratory function. Low tidal volume, positive end-expiratory pressure (PEEP), and lung recruitment maneuvers (LRMs) were recommended for patients under mechanical ventilation. However, based on the current evidence, there was no consensus on whether LRMs should be routinely used for anesthetized patients with healthy lungs, and the benefits of them remained to be determined.

**Materials and methods:**

To evaluate the benefits of LRMs on patients undergoing surgery with general anesthesia, we searched relevant studies in PubMed, EMBASE, Ovid Medline and the Cochrane Library up to June 30, 2018. The primary outcome was postoperative pulmonary complications (PPCs).

**Results:**

Twelve trials involving 2756 anesthetized patients were included. The results of our study showed a significant benefit of LRMs for reducing the incidence of PPCs (RR = 0.67; 95%CI, 0.49 to 0.90; P<0.05; Chi^2^ = 32.94, p for heterogeneity = 0.0005, I^2^ = 67%). After subgroup analyses, we found LRMs combining with lung protective ventilation strategy and sustained recruitment maneuvers were associated with reducing the occurrence of PPCs. The results also revealed that the use of LRMs improved PaO_2_/FiO_2_ in non-obese patients, but with extremely high heterogeneity (I^2^ = 95%).

**Conclusion:**

According to the findings from contemporary meta-analysis, LRMs combining with lung protective ventilation strategy may have an association with decreasing in the incidence of PPCs and improvement of oxygenation on non-obese patients. However, the conclusions must be interpreted cautiously as the outcome may be influenced dramatically due to varied LRMs and ventilation patterns.

## Introduction

General anesthesia and mechanical ventilation were broadly used in patients who underwent a wide variety of standard surgical procedures. However, emerging evidence showed that respiratory function would be impaired during mechanical ventilation because of decreasing functional residual capacity (FRC), atelectasis and even mechanical ventilator-associated lung injury [1–3]. A lot of researchers devoted their energies to finding the effective strategies to protect lung tissue including low tidal volume, positive-end-expiratory-pressure (PEEP) and lung recruitment maneuvers (LRMs) [4]. The purpose of them was to minimize the size of unavailable lung area, avoid atelectasis, and prevent lung over-extension [5, 6]. Previous evidence had proven low tidal ventilation played a pivotal role in lung protective function in anesthetized patients [7], while the effectiveness data of other strategies remained to be determined.

Recruitment maneuvers, as the most controversial among all, had been widely studied. Despite the availability of a variety of recruitment maneuvers, such as transient elevation in driving pressure or staircase elevation until peak pressure maintaining at 40–45 cmH_2_O for recruiting collapsed alveolar, the usefulness of which was still not fully understood. No guidelines were developed for LRMs so far owing to lack of high-quality randomized controlled trials (RCTs). In previous studies, the benefits of LRMs were reported in improving lung compliance and oxygenation on the patients underwent cesarean section in general anesthesia [8]. While a recent multicenter trial presented that routinely using lung recruitment maneuver had no beneficial effects on patients underwent general anesthesia [9]. In summary, based on the currently available evidence, there was no consensus on whether LRMs should be routinely used in anesthetized patients without lung diseases.

Thus, we carried out a meta-analysis of randomized controlled trials (RCTs) to evaluate the validity of LRMs in reducing postoperative pulmonary complications (PPCs) and improving arterial oxygen partial pressure/fractional inspired oxygen (PaO_2_/FiO_2_) for patients underwent surgery with general anesthesia.

## Materials and methods

In a meta-analysis, both ethical approval and patient consent are waived. This meta-analysis has been registered on https://www.crd.york.ac.uk/prospero/ with the registration number CRD42018106510.

### Literature review and search strategy

According to the recommendations from the Cochrane Handbook for Systematic Reviews of Interventions statement, Preferred Reporting Items for Systematic Reviews and Meta-Analyses statement (PRISMA) guidelines, PubMed, Embase, Ovid Medline and Cochrane library were searched without language restrictions from inception to June 30, 2018.

MeSH terms, keywords and various combinations relevant to general anesthesia were used to perform the search. The terms used included ‘anesthesias general’ or ‘general anesthesia’ or ‘general anesthesias’ or ‘anaesthesia general’ or ‘anesthesia general’ or ‘anesthesia general’ or ‘general anaesthesia’. There were no MeSH terms relevant to LRMs, so the search items used were ‘lung recruitment maneuver’ or ‘recruitment manoeuver’ or ‘lung volume recruitment’ or ‘lung recruitment’ or ‘recruit manoeur’ or ‘recruit manouev’ or ‘recruit maneuv’ or ‘recruit manuev’ on the basis of the previous article[10]. Then, we combined the above results with the RCT's MeSH and its related words to get the results.

### Criteria for considering studies for this review

#### Types of studies

RCTs which were associated with the above items were retrieved. Because of inappropriate for meta-analysis, we excluded the cross-over trials.

#### Types of participants

Inclusion criteria were: (1) Population: adults (≥18 years) without previous lung disease and undergoing mechanical ventilation in general anesthesia; (2) Intervention: using LRMs; (3) Comparison: non-LRMS; (4) Outcomes: PPCs; (5) Design: prospective RCTs.

#### Types of interventions

The studies that compared LRMs and non-LRM in anesthetized patients were included. The LRMs techniques were defined as any stepwise or sustain maneuvers elevating airway pressure to avoid atelectasis and maintain the open-status of alveolar. We defined non-LRM as any mechanical ventilation patterns without LRMs, including combined with PEEP or not.

#### Type of outcome measures

Based on the study protocol, the primary end-point was the incidence of PPCs. PPCs were defined as a composite of complications occurring during the hospital stay, including hypoxemia, bronchospasm, pulmonary infection, pulmonary infiltrate, aspiration pneumonia, acute respiratory distress syndrome (ARDS), atelectasis, pleural effusion, pulmonary edema, and pneumothorax. The secondary outcome was the PaO_2_/FiO_2_ ratio. The PaO_2_/FiO_2_ ratio was defined as data at the end of surgery, pre-extubation, immediately after extubation or in PACU. If the multi-measurement were reported in the individual study, we would choose an earlier time, i.e., right after the surgery. Obviously, the primary outcome must be reported by qualified articles.

### Data selection

We (Yu Cui, Rong Cao) sequentially reviewed all titles, abstracts, and then full texts. Later, we determined enrolled trials by assessing eligibility and outcomes independently. Disagreements were settled by discussion. If necessary, the third reviewer (Tian-qing Gong) was engaged and adjudicated. Duplicate reports, non-randomized controlled trials, case reports, reviews, pediatric, and non-human articles were abolished. Additionally, conference abstracts and study protocols were also excluded unless published as full-text reports.

Two investigators (Yu Cui, Rong Cao) collected the related data as follows: first author, year of publication, study design, sample size, age, BMI (kg/m^2^), surgical procedure, intervention LRMs, intervention tidal volume, intervention description, control description and PPCs occurring in the first 7 days after surgery, as well as PaO_2_/FiO_2_.

One reviewer (Yu Cui) imported the data and the other one (Rong Cao) double-checked for data accuracy.

### Quality assessment

Cochrane Collaboration Risk of Bias, a classical and widely used quality assessment tool, was utilized for quality assessment. This tool included random sequence generation, allocation concealment, performance bias, detection bias, attribution bias, reporting bias and others. According to the instruction, the risk of bias was judged on three levels (low, unclear and high risk of bias) from the aforementioned above seven parts based on the original studies. GRADEpro [11], as an approach to grading the quality of evidence and strength of recommendations for strategies, also was used to rate the quality of evidence.

Risk of bias analysis was performed by Review Manager Version 5.3 for Windows (RevMan, The Cochrane Collaboration, Oxford, United Kingdom) and GRADEpro (McMaster University, Hamilton, ON, 2014) respectively for the accuracy of the assessment.

### Statistical analysis

Continuous variables and dichotomous variables were extracted with mean ± standard deviations (SDs) and numbers. For dichotomous outcomes, we calculated the odds ratios (ORs) or risk ratios (RRs) with 95% confidence intervals (CIs). Mean differences (MDs) with 95% CIs were calculated for continuous outcomes. If medians (IQR) were reported and the sample size was large enough, we could consider median as equal to the mean and SD equal to IQR/1.35 [12]; otherwise, the data were excluded. The Mantel-Haenszel and Inverse-Variance tests were used to analyze dichotomous outcomes and continuous variables among pooled studies, respectively. I^2^ value was considered as an indicator of heterogeneity, which was the evidence of statistically significant heterogeneity while the amount higher than 50%.

Heterogeneity assumption was also measured by p-value. P ≤ 0.10 indicated statistical significance in heterogeneity, and the random-effects model was selected for statistical analysis; otherwise, the fixed-effects model was selected when p>0.10. If p-value was still lower than 0.10 in the random effects model, a sensitivity analysis was conducted by removing each study orderly and re-analysis again to distinguishing potential high influence studies. The subgroup analysis was performed for further evaluation according to different surgical procedures, enrolled age, BMI, the level of PEEP, and tidal volume in order to find a substantial reason for significant heterogeneity.

Confidence intervals (CI) were calculated and presented in Forest plots. Funnel plots analysis was performed when the number of enrolled studies was up to ten. Publication bias was evaluated by Egger’s test and Begg’s test using in the incidence of PPCs. Review Manager 5.3 (RevMan, The Cochrane Collaboration, Oxford, United Kingdom), GRADEpro (McMaster University, Hamilton, ON, 2014) and Stata version14.0 (StataCorp) were applied for statistical analyses.

## Results

### Description of studies

**Fig 1:**
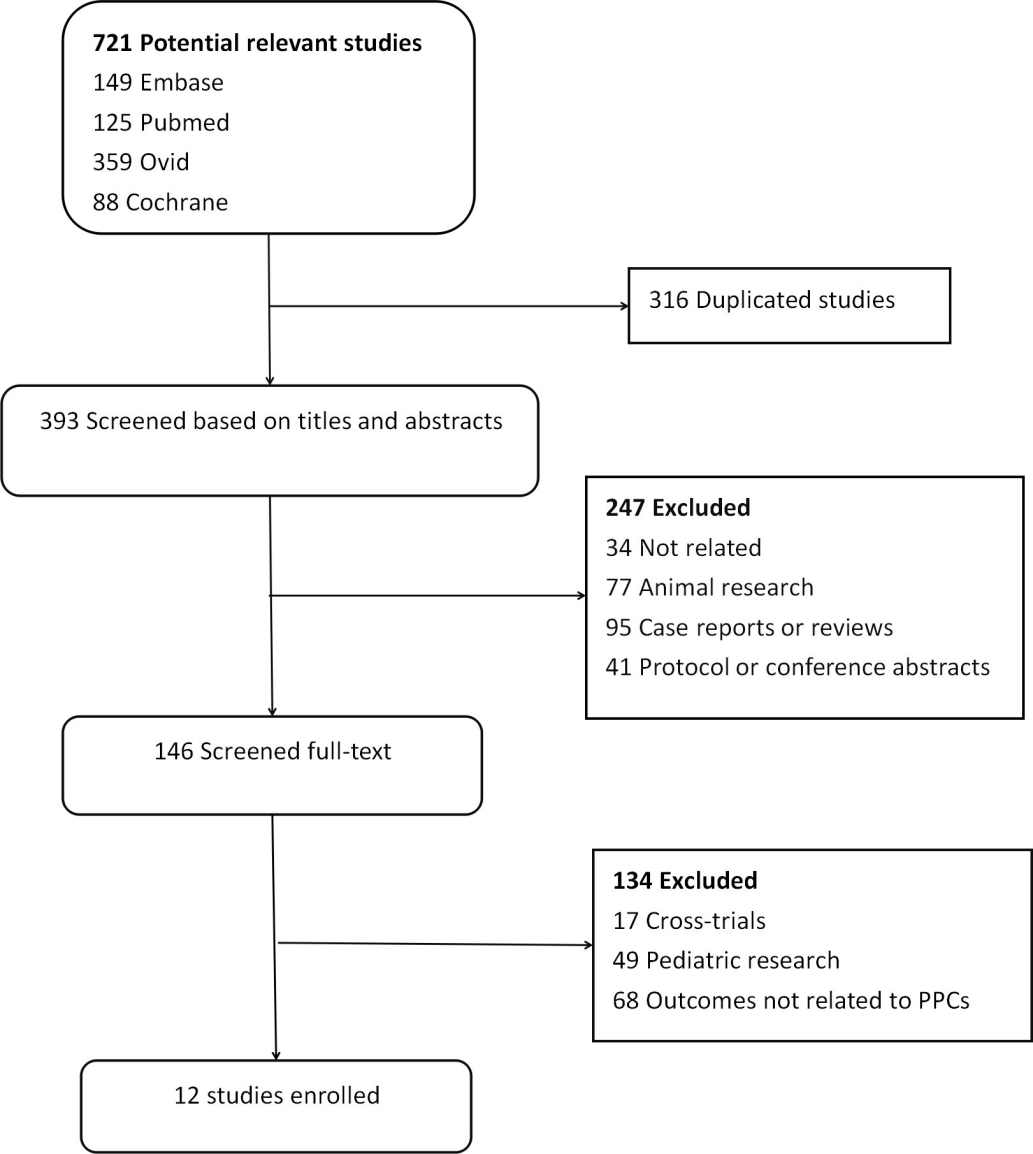
Flow chart of selecting process about this meta-analysis.

The process of literature screening was listed in Fig 1. We identified 721 potentially relevant studies (Pubmed 125, Embase 149, Medline 359, Cochrane library database 88, other resources 0). After careful selection, 709 articles did not meet the inclusion criteria, such as duplicated publications (316), animal research (77), case report or reviews (95), protocol or conference abstracts (41), cross-trials (17), pediatric research (49) and unrelated (102). Finally, 12 RCTs with 2,756 patients were pooled [8, 9, 13–22]. The basic study characteristics, controlled ventilation mode, as well as intervention LRMs in enrolled studies, were described in Table 1. All the twelve trials reported the incidence of PPCs and gave detailed data on each complication. In overall analysis, we realized most trials selected the patients undergoing abdominal or pelvic surgery, except one on spinal surgery [13].

**Table 1:** Basic characteristics about enrolled studies.

Study Characteristics						Intervention maneuver			Control maneuver		PPCs definition	PPCs endpoint
First author, year	Centers (n)	Patients (n)	Age (year)	BMI (kg/m^2^)	Surgery procedure	Lung recruitment maneuver	Tidal volume (ml/kg)	PEEP (cmH_2_O)	Tidal volume (ml/kg)	PEEP (cmH_2_O)		
Ferrando,2018[9]	21	967	≥18	<35	abdominal surgery	step-wise until airway pressure reached 40 cmH_2_O; performed after intubation, repeat according to patient’s requirement	8	Individualised PEEP	8	5	pneumonitis, atelectasis, dyspnoea, hypoxaemia, pneumothorax, pneumonia, ARDS and so on	the first 7 postoperative days
Nestler,2017[15]	1	50	≥18	≥35	laparoscopic surgery	peak pressure 50cm H_2_O, PEEP 30cm H_2_O, respiratory rate 6 bpm, for 10 cycles an RM followed by a decremental PEEP titration and an additional RM was performed before extubation	8	Individualised PEEP	8	5	pneumonia or the need for invasive or non-invasive ventilation	During hospital stay
Choi,2017[19]	1	60	60–80	≤31	RARP	Staircase PEEP (4-16cmH_2_O), performed after intubation	6–8	5	6–8	5	Atelectasis or decreased saturation	During hospital stay
Aretha,2016[8]	1	90	>18	18–44	cesarean section	Staircase PEEP (0-20cmH_2_O) until a plateau pressure 45 cmH_2_O LRM lasted about 3 min and was not repeated	6	8	8	0	Pneumonia or pulmonary embolism	Postoperative day 3
Pi X,2015[16]	1	63	≥60	<35	non-laparoscopic abdominal elective major surgery	The tidal volume was increased 4 ml/kg until plateau pressure of 30 cm H_2_O three times; recruitment maneuvers were performed in every 30 min after tracheal intubation	7	8	7/9	8/0	dyspnea, pneumonia, pneumothorax, respiratory distress and chronic respiratory failure	During hospital stay
Shen, 2015[22]	1	120	Adult	NM	Thoracic or abdominal surgery	Applying a continuous positive airway pressure of 30cmH2O for 30s; recruitment maneuvers were performed in every 30 min after tracheal intubation	6	6	10	0	Pulmonary infection or atelectasis	The first 7 postoperative days
Hemmes,2014[14]	30	900	≥18	<40	open abdominal surgery	incremental increases in tidal volume; recruitment manoeuvres were performed after induction of anaesthesia, after any disconnection from the ventilator, and just before tracheal extubation	8	12	8	2	hypoxemia, bronchospasm, pulmonary infection, aspiration pneumonitis, ARDS, atelectasis, pulmonary edema, pneumothorax	The first 5 postoperative days
Ge Y,2013[13]	1	60	70–85	Not mention	spinal fusion surgery	PIP = 45cmH_2_O, Pplat≤30-35cmH_2_O; recruitment maneuvers repeated every 15 min	6	10	10	0	pulmonary infection, atelectasis, respiratory failure, hypoxemia	The first postoperative day
Futier E, 2013[20]	7	200	≥40	<35	Laparoscopic or non-laparoscopic elective major abdominal surgery	Applying a continuous positive airway pressure of 30cmH_2_O for 30s; recruitment maneuvers repeated every 30 minutes after tracheal intubation	6–8	6–8	10–12	0	Pneumonia or the need for invasive or noninvasive ventilation for acute respiratory failure	The first 7 postoperative days
Weingarten,2010[17]	1	40	>65	≤35	major open abdominal surgery	Staircase PEEP (0-20cmH_2_O) Lung recruitment was repeated at 30 and 60 min after the first recruitment and hourly thereafter.	6	12	10	0	acute lung injury, non-cardiogenic pulmonary oedema, pneumonia, atelectasis, pneumothorax	In the recovery room
Whalen,2006[18]	1	20	25–65	>40	laparoscopic bariatric surgery	Staircase PEEP (0-20cmH_2_O), the peak pressure not exceeding 50cmH_2_O; the requirement for repeated recruitment depended on the Pao2 response to the preceding maneuver		12	8,	4	pulmonary embolism, respiratory failure requiring mechanical ventilation or delayed tracheal extubation, pneumonia, atelectasis	During hospital stay

BMI: Body mass index; RARP: Robotic-assisted laparoscopic radical prostatectomy; PEEP: Positive end-expiratory pressure; PIP: peak inspiratory pressure; ARDS: acute respiratory distress syndrome

### Quality assessment

**Fig 2:**
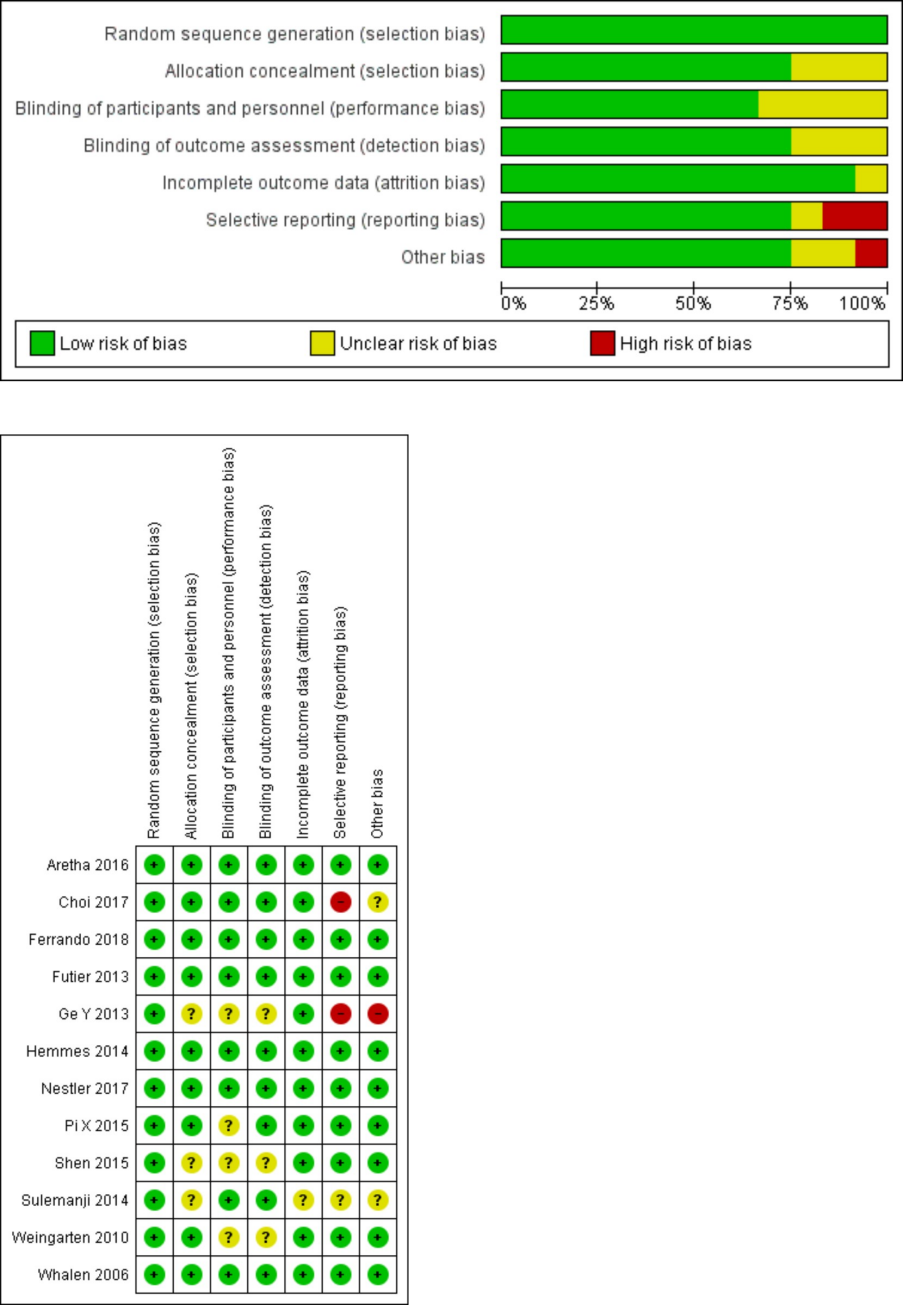
Assessment of risk bias for RCTs: (A) a graph with percentages for all included studies; (B) a summary of bias for each included study.

Quality assessment by Review manager 5.3 and GRADEpro was shown in Fig 2A and 2B, Table 2. All of them presented a low risk of random sequence generation and nine out of 12 studies showed a low risk of allocation concealment by describing the randomized method in detail [8,9,14–20]. Four out of 12 RCTs were not blinded to investigators, and the outcomes may be influenced by the lack of blinding [13,16, 17, 22]. No evidence of publication bias was detected by the Egger’s test (P = 0.297) and the Begg’s test (P = 0.373) in the incidence of PPCs with STATA (version 15.1).

**Table 2:** Quality of evidence by GRADE.

Certainty assessment							№ of patients		Effect		Certainty	Importance
№ of studies	Study design	Risk of bias	Inconsistency	Indirectness	Imprecision	Other considerations	post-operative pulmonary complcations	placebo	Relative (95% CI)	Absolute (95% CI)		
**complications**												
10	randomised trials	serious ^a^	not serious	not serious	not serious	none	415/1361 (30.5%)	511/1395 (36.6%)	**RR 0.66** (0.49 to 0.90)	**125 fewer per 1,000** (from 187 fewer to 37 fewer)	⨁⨁⨁◯ MODERATE	
**complications—elder patients**												
4	randomised trials	serious ^b^	not serious	not serious	not serious	none	13/99 (13.1%)	36/122 (29.5%)	**RR 0.39** (0.22 to 0.68)	**180 fewer per 1,000** (from 230 fewer to 94 fewer)	⨁⨁⨁◯ MODERATE	
**complications—Non-elder patients**												
7	randomised trials	serious ^a^	not serious	not serious	serious ^a^,^c^	none	398/1252 (31.8%)	471/1266 (37.2%)	**RR 0.86** (0.77 to 0.95)	**52 fewer per 1,000** (from 86 fewer to 19 fewer)	⨁⨁◯◯ LOW	
**complications—obese**												
2	randomised trials	serious ^d^	not serious	not serious	not serious	none	5/35 (14.3%)	2/35 (5.7%)	**RR 1.94** (0.49 to 7.74)	**54 more per 1,000** (from 29 fewer to 385 more)	⨁⨁⨁◯ MODERATE	
**complications—Non-obese**												
8	randomised trials	serious ^a^	not serious	not serious	not serious	none	387/1053 (36.8%)	432/1093 (39.5%)	**RR 0.91** (0.81 to 1.02)	**36 fewer per 1,000** (from 75 fewer to 8 more)	⨁⨁⨁◯ MODERATE	
**complications—LRMs+Non-Individual PEEP**												
10	randomised trials	serious ^a^	not serious	not serious	not serious	none	201/584 (34.4%)	212/615 (34.5%)	**RR 0.65** (0.46 to 0.91)	**121 fewer per 1,000** (from 186 fewer to 31 fewer)	⨁⨁⨁◯ MODERATE	
**complications—LRMs with Individual PEEP**												
2	randomised trials	not serious	not serious	not serious	not serious	none	191/504 (37.9%)	222/513 (43.3%)	**RR 0.88** (0.76 to 1.02)	**52 fewer per 1,000** (from 104 fewer to 9 more)	⨁⨁⨁⨁ HIGH	
**complications-LRMs compare to ZEEP**												
6	randomised trials	serious ^a^	not serious	not serious	not serious	none	37/372 (9.9%)	80/372 (21.5%)	**RR 0.51** (0.25 to 1.05)	**105 fewer per 1,000** (from 161 fewer to 11 more)	⨁⨁⨁◯ MODERATE	
**complications-LRMs compare to different level of PEEP**												
5	randomised trials	serious ^a^	not serious	not serious	not serious	none	372/963 (38.6%)	400/978 (40.9%)	**RR 0.94** (0.84 to 1.05)	**25 fewer per 1,000** (from 65 fewer to 20 more)	⨁⨁⨁◯ MODERATE	
**complications-LRMs compare to low tidal volume**												
8	randomised trials	serious ^a^	not serious	not serious	not serious	none	380/1051 (36.2%)	417/1085 (38.4%)	**RR 0.92** (0.79 to 1.07)	**31 fewer per 1,000** (from 81 fewer to 27 more)	⨁⨁⨁◯ MODERATE	
**complication-LRMs compare to high tidal volum**												
4	randomised trials	serious ^a^	not serious	not serious	not serious	none	35/310 (11.3%)	94/310 (30.3%)	**RR 0.66** (0.49 to 0.90)	**103 fewer per 1,000** (from 155 fewer to 30 fewer)	⨁⨁⨁◯ MODERATE	
**complication-stepwise LRMs**												
7	randomised trials	serious ^a^	not serious	not serious	not serious	none	379/1034 (36.7%)	421/1068 (39.4%)	**RR 0.91** (0.77 to 1.06)	**35 fewer per 1,000** (from 91 fewer to 24 more)	⨁⨁⨁◯ MODERATE	
**complication-sustain LRMs**												
4	randomised trials	serious ^a^	not serious	not serious	not serious	none	32/315 (10.2%)	86/315 (27.3%)	**RR 0.37** (0.21 to 0.66)	**172 fewer per 1,000** (from 216 fewer to 93 fewer)	⨁⨁⨁◯ MODERATE	

**CI:** Confidence interval; **OR:** Odds ratio; **MD:** Mean difference

Explanations

a. because of different LRMs, and enrolled patients varied greatly.

b. different surgical procedure and fluid therapy

c. different frequency of LRMs

d. small sample siz

### Primary outcomes

#### The incidence of PPCs

**Fig 3:**
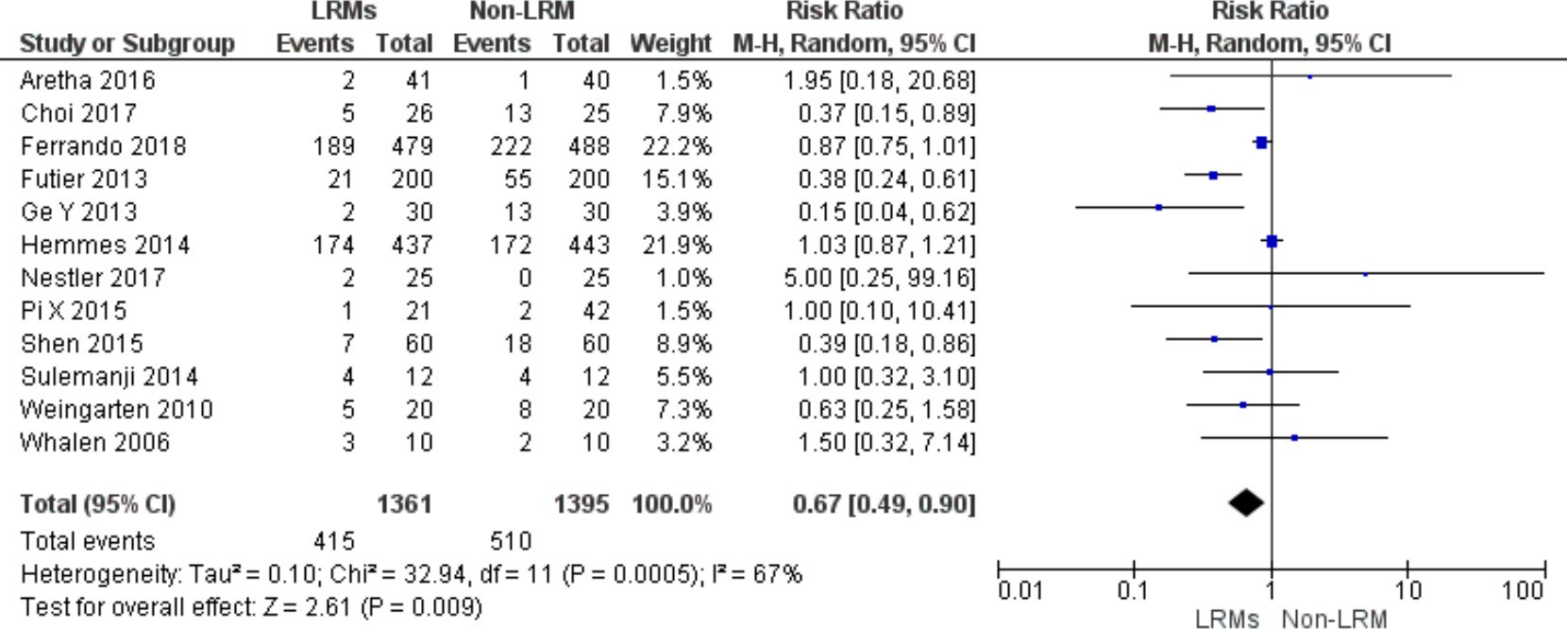
Forest plot showing for the over-all incidence of PPCs between the LRMs and non-LRM groups.

Ten RCTs including 2,756 patients reported data about the number of patients with PPCs with an overall incidence of 33.6% (415/1361 in LRMs group, 511/1395 in non-LRM group). LRMs were superior than non-LRM in reducing the incidence of PPCs using the random effect model (RR = 0.67; 95%CI, 0.49 to 0.90; p = 0.007), with high heterogeneity (Chi^2^ = 32.94, p for heterogeneity = 0.0005, I^2^ = 67%) (Fig 3).

#### Subgroup analysis according to co-intervention with the individual PEEP strategy

**Fig 4:**
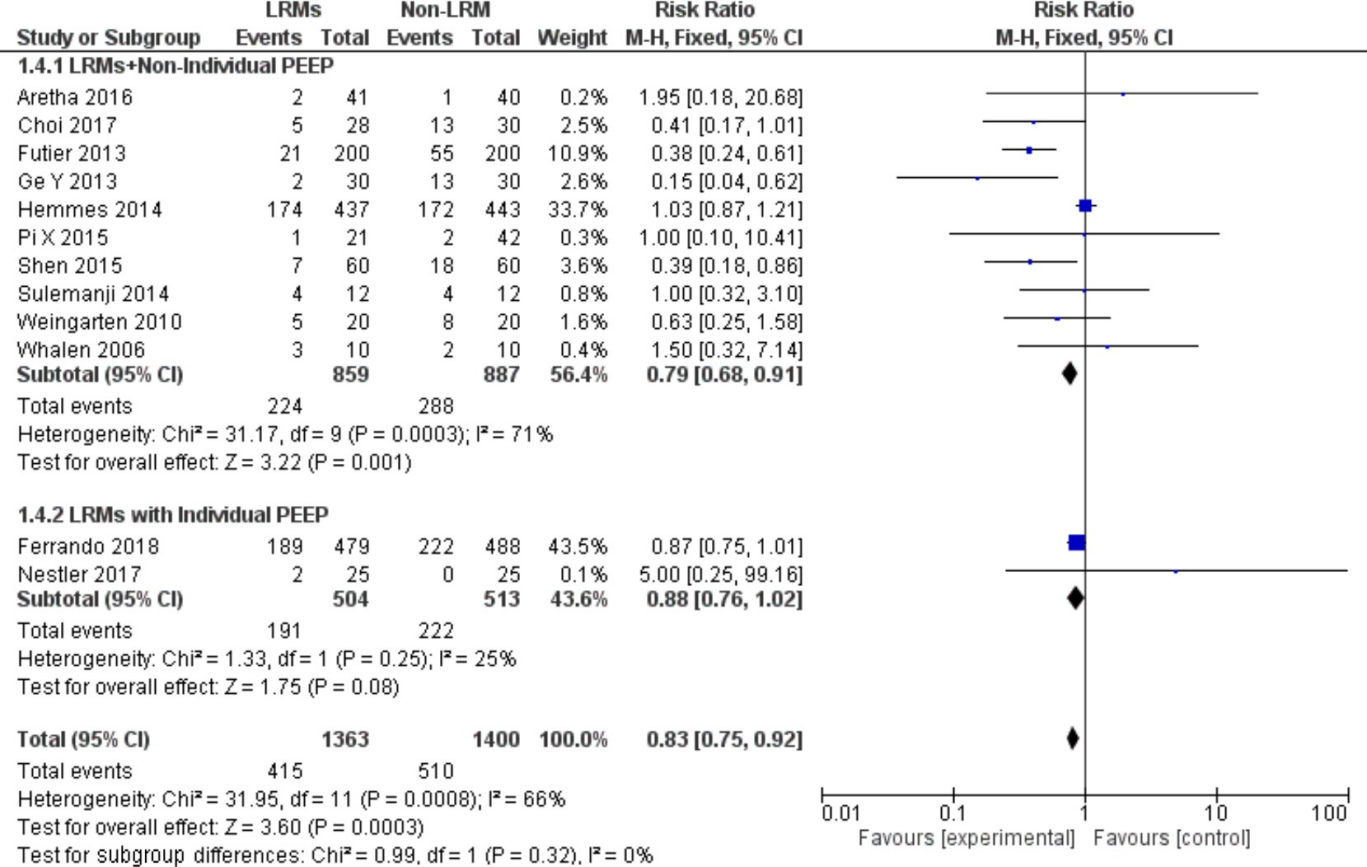
Forest plot for subgroup analysis of the incidence of PPCs between the LRMs and Non-LRM groups: According to co-intervention with individual PEEP or not.

Two of 12 trials combined with an individual PEEP ventilation strategy as a co-intervention with LRMs [9, 15]. The co-intervention with individual PEEP and LRMs did not generate synergistic effects, and there was no significant reduction of the incidence of PPCs (RR = 0.88; 95%CI, 0.76to 1.02; p = 0.08; Chi^2^ = 1.33, p for heterogeneity = 0.25, I^2^ = 25%) (Fig 4).

#### Subgroup analysis according to design control group as non-LRM without PEEP or with low PEEP

**Fig 5:**
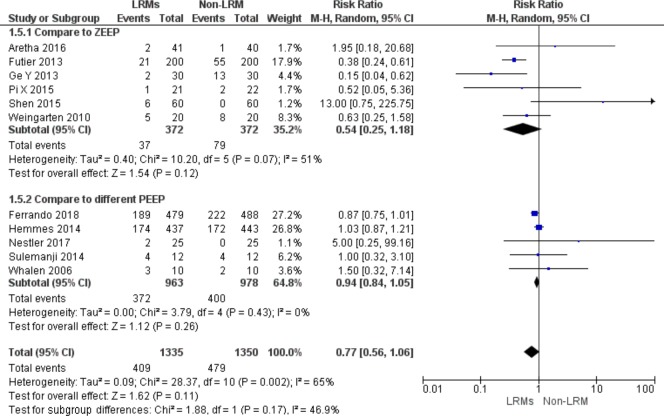
Forest plot for subgroup analysis of the incidence of PPCs between the LRMs and Non-LRM groups: According to design control group as non-LRM without PEEP or low PEEP.

Six out of 12 trials including 744 patients designed non-LRM without PEEP as control group [8,13,16,17,20,22] and five studies with low PEEP [9,14,15,18,21]. However, the results demonstrated that there was no significant reduction of the incidence of PPCs, regardless of PEEP value (RR = 0.54; 95%CI, 0.25 to 1.18; P = 0.12; Chi^2^ = 10.20, p for heterogeneity = 0.07, I^2^ = 51%) (RR = 0.94; 95%CI, 0.84 to 1.05; P = 0.26; Chi^2^ = 3.79, p for heterogeneity = 0.43, I^2^ = 0%)(Fig 5).

#### Subgroup analysis according to design control group as non-LRM with different tidal volume

**Fig 6:**
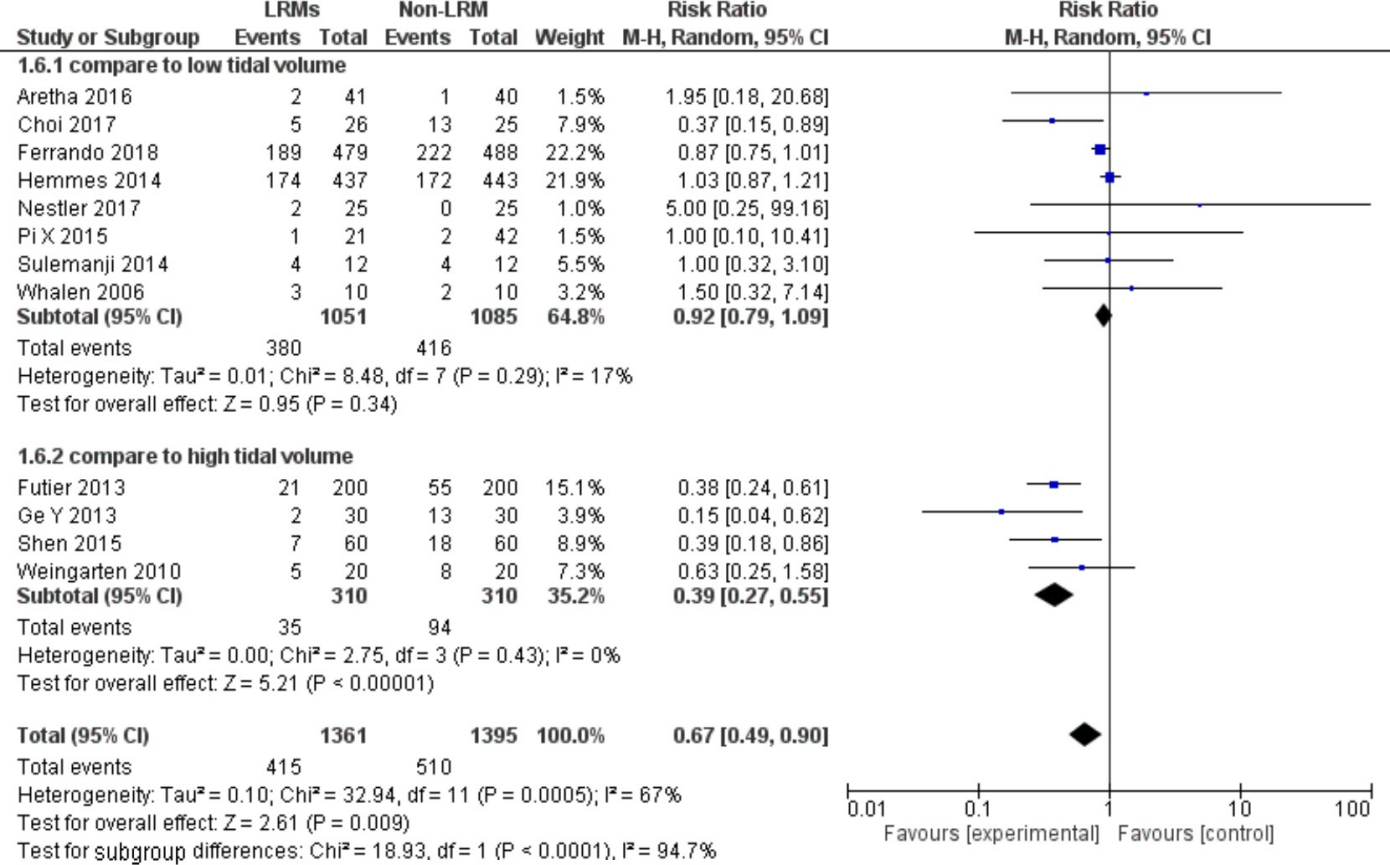
Forest plot for subgroup analysis of the incidence of PPCs between the LRMs and Non-LRM groups: According to design control group as non-LRM with different tidal volume.

Four out of 12 studies included 620 patients compared to non-protective lung ventilation with tidal volume 10ml/kg [13,17,20,22]. Compared patients with high tidal volume(10 ml/kg or above), LRMs with low tidal volume (6–8 ml/kg) could reduce the incidence of PPCs dramatically with low heterogeneity (RR = 0.39; 95%CI, 0.27 to 0.55; P = 0.43; Chi^2^ = 2.75, p for heterogeneity = 0.43, I^2^ = 0%) (Fig 6). However, compared patients with low tidal volume, LRMs with low tidal volume could not reduce the incidence of PPCs (RR = 0.92; 95%CI, 0.79 to 1.09; P = 0.34; Chi^2^ = 8.48, p for heterogeneity = 0.29, I^2^ = 17%) (Fig 6).

#### Subgroup analysis according to a different type of LRMs

**Fig 7:**
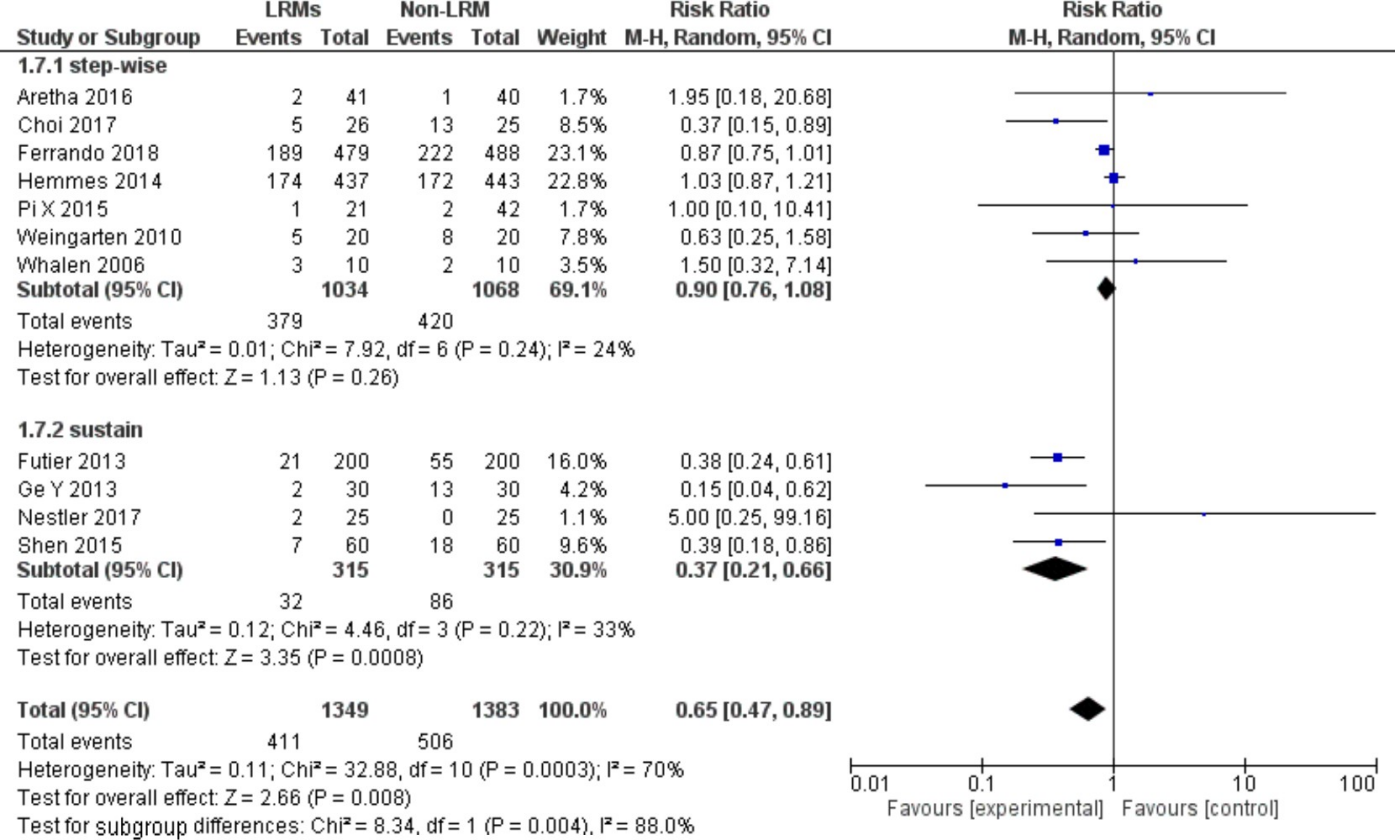
Forest plot for subgroup analysis of the incidence of PPCs between the LRMs and Non-LRM groups: According to different type of LRMs.

We did the subgroup analysis according to a different type of recruitment maneuver (i.e., sustain vs. step-wise cycling maneuver). For patients using step-wise cycling maneuver, there was no significant difference between two groups on the incidence of PPCs (RR = 0.90, 95%CI, 0.76 to 1.08; P = 0.26; p for heterogeneity = 0.24, I^2^ = 24%, Fig 7). For sustain maneuver, LRMs could reduce the incidence of PPCs but with moderate heterogeneity (RR = 0.37, 95%CI, 0.21 to 0.66; P = 0.0008; p for heterogeneity = 0.22, I^2^ = 33%, Fig 7).

#### Subgroup analysis according to age

**Fig 8:**
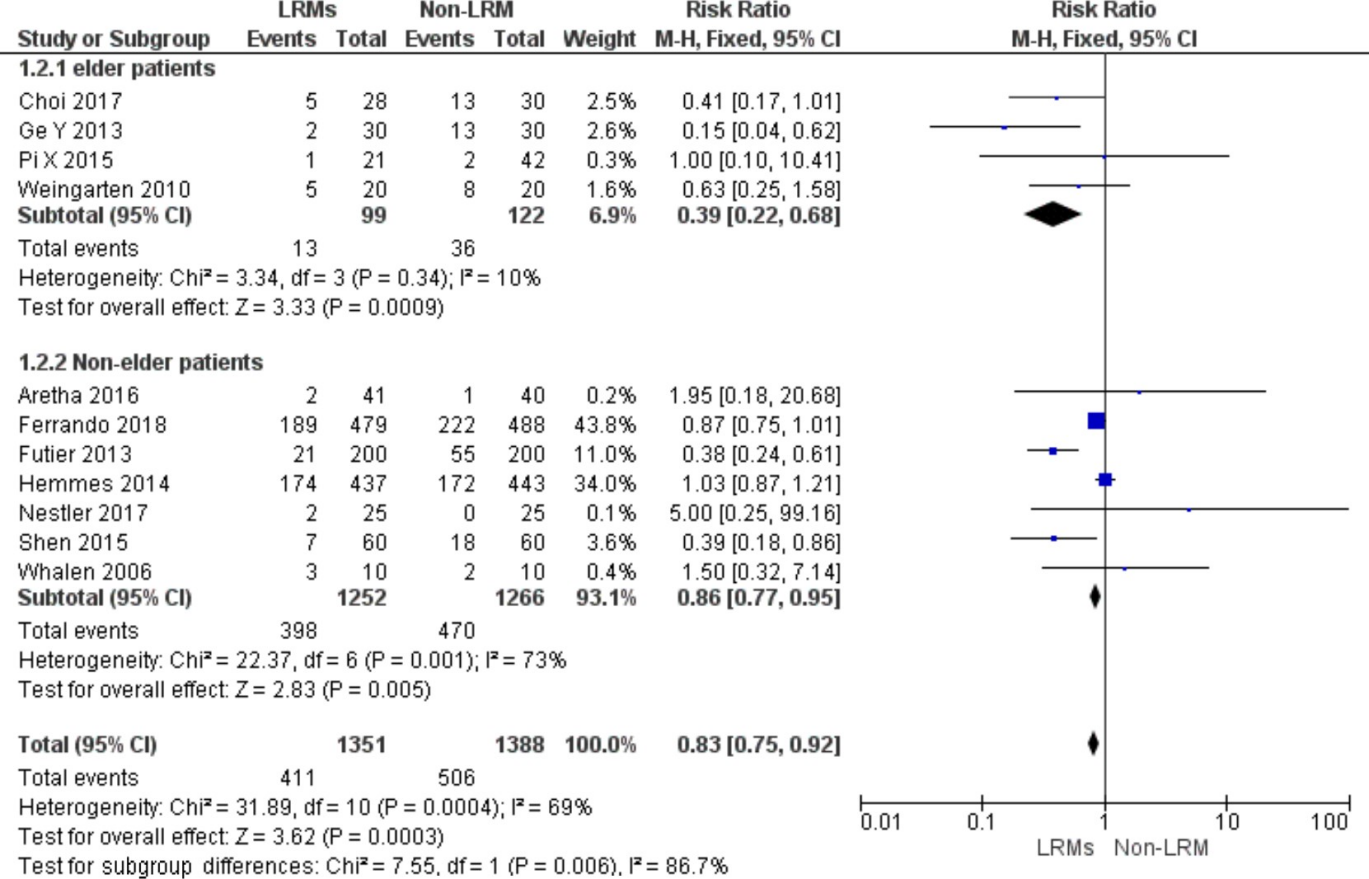
Forest plot for subgroup analysis of the incidence of PPCs between the LRMs and Non-LRM groups: According to difference age: ≥ 60 years or others.

Four out of 12 studies included 221 patients aged over 60 years [13,16,17,19]. For elderly patients, LRMs could reduce the incidence of PPCs remarkably either in elderly or non-elderly patients (RR = 0.39; 95%CI, 0.22 to 0.68; P = 0.0009; Chi^2^ = 3.34, p for heterogeneity = 0.34, I^2^ = 10%) vs. (RR = 0.86, 95%CI, 0.77 to 0.95; P = 0.005; p for heterogeneity = 0.001, I^2^ = 73%, Fig 8).

#### Subgroup analysis according to BMI

**Fig 9:**
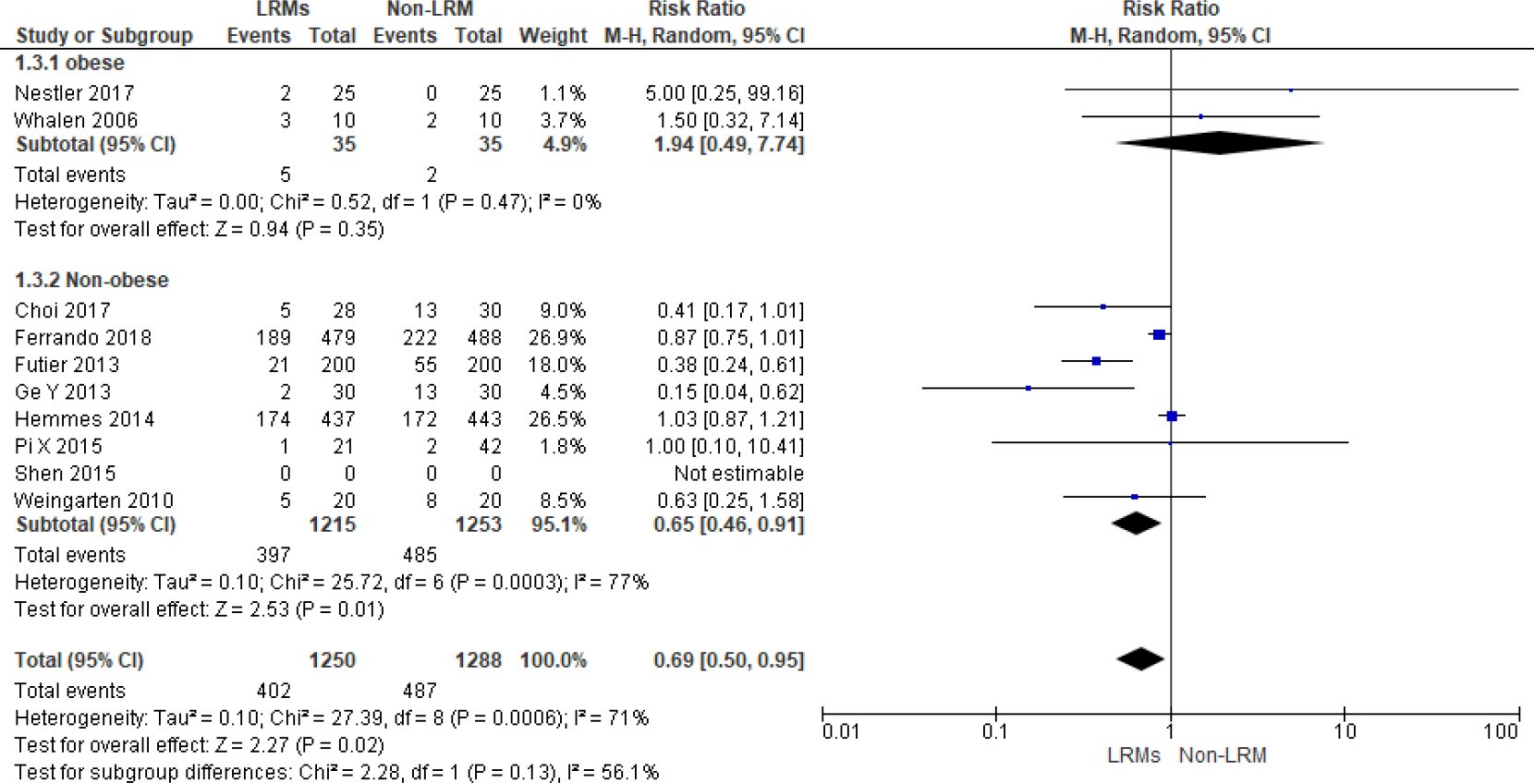
Forest plot for subgroup analysis of the incidence of PPCs between the LRMs and Non-LRM groups: According to BMI: ≥ 35 kg/m^2^ or others.

Two out of 12 trials including 70 patients focused on obese patients with BMI>35kg/m^2^ [15, 18]. For non-obese patients, LRMs could reduce the incidence of PPCs remarkably with the random effect model, but there was no significant difference in obese population (RR = 0.65; 95%CI, 0.46 to 0.91; P = 0.01; Chi^2^ = 25.72, p for heterogeneity = 0.0003, I^2^ = 76% vs. RR = 1.94, 95%CI, 0.49 to 7.74; P = 0.35; Chi^2^ = 0.52, p for heterogeneity = 0.47, I^2^ = 0%, respectively, Fig 9).

### Secondary outcomes

**Fig 10:**
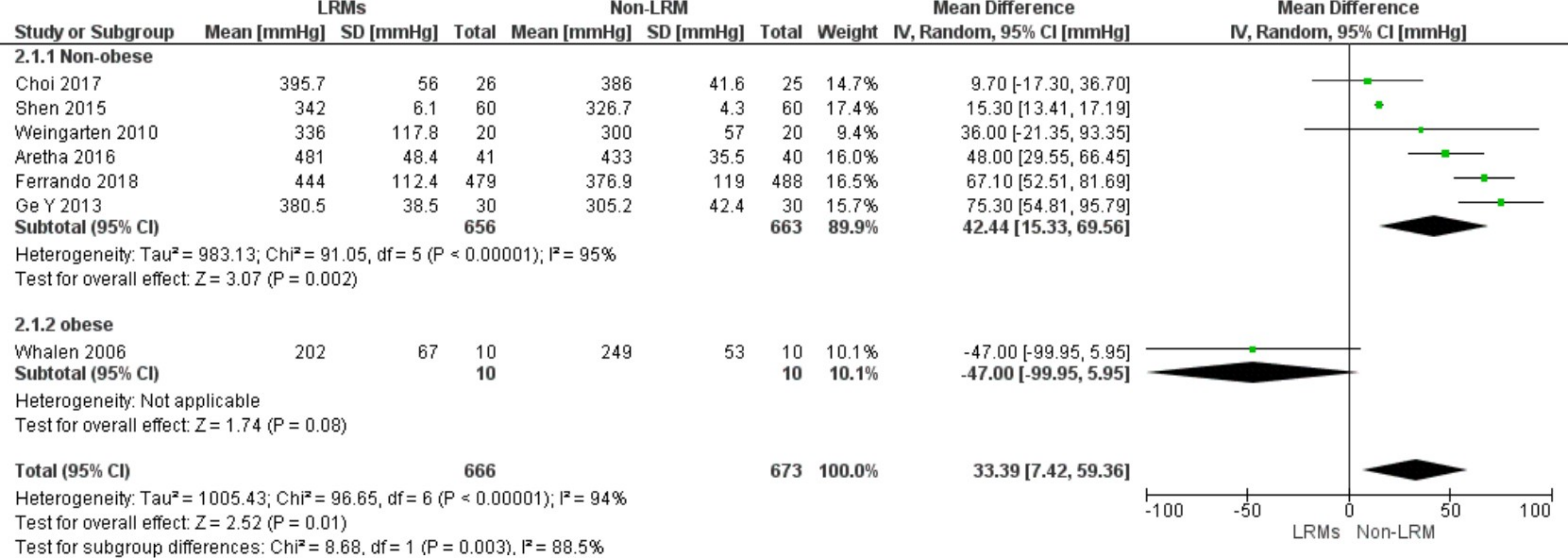
Forest plot showing the subgroup comparison of PaO_2_/FiO_2_ between the LRMs and Non-LRM groups: According to BMI: ≥ 35 kg/m^2^ or others.

Six trials presented PaO_2_/FiO_2_ ratio at different time-points, such as pre-extubation, at the end of surgery or in PACU. The PaO_2_/FiO_2_ ratio varied considerably among the research. LRMs improved oxygenation in non-obese patients significantly (six trials; MD 42.4 mmHg; 95% CI, 15.3–69.6 mmHg, P<0.01, Fig 10), but without benefit in obese patients.

## Discussion

We pooled 12 trials that presented the primary outcomes in this meta-analysis. This meta-analysis of from RCTs comparing LRMs with non-LRM in anesthetized patients who underwent abdomen, pelvic and spinal surgeries. The result suggested that the overall incidence of PPCs in LRMs group was significantly lower than the non-LRM group. However, the result with high heterogeneity was not convinced. After carefully review, we found that there were four studies which compared with non-protective lung ventilation group [13,17,20,22], and six studies compared to control group without PEEP [8,13,16,17,20,22]. To our knowledge, lung protective ventilation strategy, including low tidal volume and PEEP, was an integral part of mechanical ventilation management. Therefore, studies those compared with non-protective lung ventilation strategy was at high risk of bias. To reduce bias, we performed subgroup analyses according to different co-interventions and type of maneuver.

After subgroup analyses, we found that LRMs with PEEP was not superior to non-LRMs with low or zero PEEP on the incidence of PPCs. In the light of present clinical knowledge, most of the anesthesia providers preferred applying PEEP as a lung protective method. Based on the above results, we had enough evidence to suspect that the same level of PEEP was not related to lung protective effects. Indeed, protective effects of PEEP seem to be changed according to different kinds of procedures, as one study demonstrated that 5cmH_2_O PEEP in major abdominal surgery lower functional residual capacity (FRC), while the same PEEP was not associated with significant effects on FRC in craniotomy patients [23]. PEEP also could be influenced by chest wall compliance and BMI [24]. Even PEEP was also an essential component of protective ventilation regime, and there was no consensus on what the optimal PEEP was for the patients with healthy lungs undergoing general anesthesia. Studies had demonstrated the individual optimal PEEP varied widely [8, 9]. One multicenter research showed that high levels of PEEP had no relationship with preventing PPCs [14]. Of concern was the fact that excessive PEEP had been turned out to mediate lung injury and develop of PPCs [25]. Furthermore, we conducted the subgroup analysis according to utilize individual PEEP or not, and the result showed there was no difference between two groups on the incidence of PPCs. However, we could not merely get the conclusion that LRMs combining with individual PEEP was not superior to others since only two trials were enrolled [9,15], which could be expected with a high risk of bias. Regarding two pooled studies, the frequency and pressure of recruitment maneuvers varied greatly, which may immensely influence the outcomes. Moreover, the major concern was how to get the reliable value of individual optimal PEEP. Currently, the only objective technique was the titration of PEEP by esophageal manometry to detect the best pulmonary dynamic compliance. An invasive esophageal balloon and the careful assessment of compliance when PEEP was decreased were required. A recent study reported that a non-invasive electrical impedance tomography (EIT) could be used to identify optimal PEEP [26], but no EIT device was commercially available in most developing countries. Therefore, the development of new methods which could be used at the bedside with sustainable cost-effectiveness was an unmet demand.

Our results indicated that compared with the high tidal volume group, LRMs with low tidal volume could reduce the incidence of PPCs. Neto et al. had proved the dose-response relationship between tidal volume value and the risk of pulmonary complications [27], which had similar results with us. Our study also argued the notion that use of low tidal volume was unnecessary for health patients since their lung tissue change was wide-spread and may promote the development of more atelectasis [28]. Even in the population with respiratory insufficiency, the low tidal volume ventilation strategy could shorten the duration of ventilation and improve the survival rate [29]. The above evidence indicates that low tidal volume ventilation strategy may play a pivotal role in reducing the development of PPCs.

It was well known that there was no uniform operating guideline for LRMs. In clinical practice, LRMs consisted of stepwise or sustained manual inflation to a peak inspiratory pressure. A meta-analysis reported different alveolar recruitment maneuvers were equally effective in improving lung compliance for reducing PPCs [30], but the authors did not consider the heterogeneity of enrolled trials with different LRMs. After conducting subgroup analysis as among enrolled studies according to the stepwise or sustained LRMs, we found utilized sustained LRMs were associated with decreasing PPCs, while there was no benefit in patients using stepwise LRMs. Previous RCTs had shown that sustained recruitment was understood to be of value during anesthesia, but indications, as well as frequency, differed significantly among studies [13, 15, 20, 22]. The success of LRMs had a relationship with the amount of available alveolar and the characteristics of patients. Patients without lung disease would tolerant atelectasis well and not easily develop to the occurrence of PPCs.

According to definition as mentioned earlier, the list of PPCs contained hypoxemia, bronchospasm, pulmonary infection, pulmonary infiltrate, aspiration pneumonia, acute respiratory distress syndrome(ARDS), atelectasis, pleural effusion, pulmonary edema, and pneumothorax, which were associated with re-intubated, the length of mechanical ventilation, mobility and even mortality[31]. Anesthetized patients with mechanical ventilation would easily get PPCs due to the mechanical and functional changes during mechanical ventilation period, including the decrease of functional residual capacity, high inspiratory FiO_2_, ventilator-induced lung injury and the movement of the diaphragm along ventral-dorsal axis [1, 32, 33]. As a method to elevate airway pressure, LRMs were performed in order to make the collapse alveolar re-open or keep alveolar at an open state [34]. Despite both animal and clinical researches about LRMs during general anesthesia had been published [35, 36], there was a controversy about routine applying of LRMs in the way that the improvement strategy outside the intensive care unit. In our meta-analysis, the result demonstrated that LRMs could reduce the incidence of PPCs in patients. This result was similar to the previous finding of the application of LRMs could improve oxygenation in patients [37]. However, this result should be interpreted discreetly. PPCs were reported as the sum of atelectasis and desaturation in Choi’s study. It might well be that both events happened in the same patients. The number of events would be inflated as compared to the studies that define PPC at “at least one” of the events included in the definition, and the same patient would be replicated in the analysis. Besides, the study with huge bias should be treated critically. One of the enrolled studies with large sample size showed an extremely high incidence of postoperative extrapulmonary complications (55%) and pneumothorax (3%), which was unreasonable in patients without lung disease. The fluid therapy was uncontrolled, and 40% of patients received about 3000-5000ml and even more fluid. Multiple transfusion of blood products was close to 20% of patients while blood loss was 400-500ml in both groups [14]. Available evidence had shown that excessive fluid therapy was an independent risk factor of pulmonary complication [38]. Those bias should not be ignored when we interpreted the results.

Based on subgroup analyses, we demonstrated that the incidence of PPCs was reduced either in elderly or in non-elderly patients, and oxygenation was improved in non-obese patients after LRMs. However, the results must be interpreted cautiously, given that only ten obese patients were enrolled in each arm of the oxygenation related trial, which may lead to a high risk of bias. Moreover, we extracted data based on the definition of enrolled study with different criteria, as the patients with age more than 65 years were defined as elderly in some study [17], whereas 60 years in another research [16]. Besides, the non-elderly studies included two extensive studies which between them have 757 events in 1,547 patients. The first of these trials [9] enrolled patients with an average age of 65 and the second trial [14] enrolled patients with a similar average age (65 in one group; 64 in the other). All those heterogeneities may lead to unreliable results. It was well possible that a study with the inclusion criterion of patients older than 18 years effectively recruits only patients older than 80 years. The inclusion criterion, therefore, did not necessarily reflect and describe the study population very well. Consequently, we could not get the conclusion that LRMs had some benefits either in elderly patients or in non-elderly patients on reducing PPCs.

Lung function could be impaired in obese patients because high airway resistance, low pulmonary volumes, and obesity were all likely the risk factors for PPCs [39]. Although many researchers studied the application of LRMs to decrease the incidence of PPCs in obese patients, the benefits had yet to be determined. One research suggested without persistent application of PEEP, LRMs could not reduce the rate of PPCs [40]. Our study found there was no difference in the incidence of PPCs between LRMs and non-LRM group in obese patients, but this result was not convincing enough as only two studies were enrolled with small sample size and at the risk of moderate bias [15, 18]. According to the secondary outcome, we realized the PaO_2_/FiO_2_ was improved by the application of LRMs in non-obese patients, but with high heterogeneity. This may be due to the fact that each trial reported data at different time points, such as pre-extubation, end of the surgery, and in PACU. Even though as recruitment maneuver, frequency and pressure may remarkably influence the outcomes. In other words, the conclusion may be significantly varied due to different maneuvers and ventilation patterns.

### Limitation

Several limitations to this meta-analysis need to be acknowledged. There are several analyses with significant statistical heterogeneity which cannot be resolved by subgroup analyses. The quality of enrolled RCTs is another big issue as the number of the enrolled trials were small sample size. Moreover, most of the enrolled studies lack the evidence of effectiveness of LRMs on atelectasis.

## Conclusion

Recruitment maneuver was regularly performed in general anesthesia as part of a lung protective strategy to avoid lung tissue collapsed. Our meta-analysis supported that the use of recruitment maneuver may reduce the incidence of PPCs, especially combined with lung-protective ventilation strategy. Combining with individual or other levels of PEEP, there was no remarkable reduction in the incidence of PPCs. Furthermore, no consensus had been reached on the ideal recruitment strategy as the variable LRMs, despite the fact that we found sustain may be better than stepwise LRMs on the development of PPCs. Further powerful RCTs are needed to ascertain the efficacy and feasibility recruitment therapy concerning frequency, peak inspiratory pressure (PIP), optimal PEEP and suitable FiO_2_.

## Supporting information

S1 Fig PRISMA 2009 checklist. (PDF)

S2 Fig PRISMA 2009 flow diagram. (PDF)

## Abbreviations

LRMs: Lung recruitment maneuvers
PPCs: post-operative pulmonary complications
FRC: Functional residual capacity
PEEP: Positive end expiratory pressure
ARDS: Acute respiratory distress syndrome
RCTs: Randomized controlled trials
PRISMA: Preferred Reporting Items for Systematic Reviews and Meta-Analyses statement
CENTRAL: Cochrane Central Register of Controlled Trials
SD: Standard deviations
OR: Odds ratio
RR: Risk ratio
GRADE: Grades of Recommendation, Assessment, Development, and Evaluation
PACU: Post-anesthesia care unit
PIP: Peak inspiratory pressure
IQR: Interquartile range
CI: Confidence interval
MD: Mean differences
RARP: Robotic Assisted Radical Prostatectomy
