# Supplementary material for: The effect of lung recruitment maneuvers on post-operative pulmonary complications for patients undergoing general anesthesia: A meta-analysis
Source: PLoS One. 2019 May 29;14(5):e0217405. doi: 10.1371/journal.pone.0217405 (PMC6541371; doi:10.1371/journal.pone.0217405)
Supplement: S2 Fig — (PDF) [file pone.0217405.s002.pdf]

PRISMA 2009 Flow Diagram

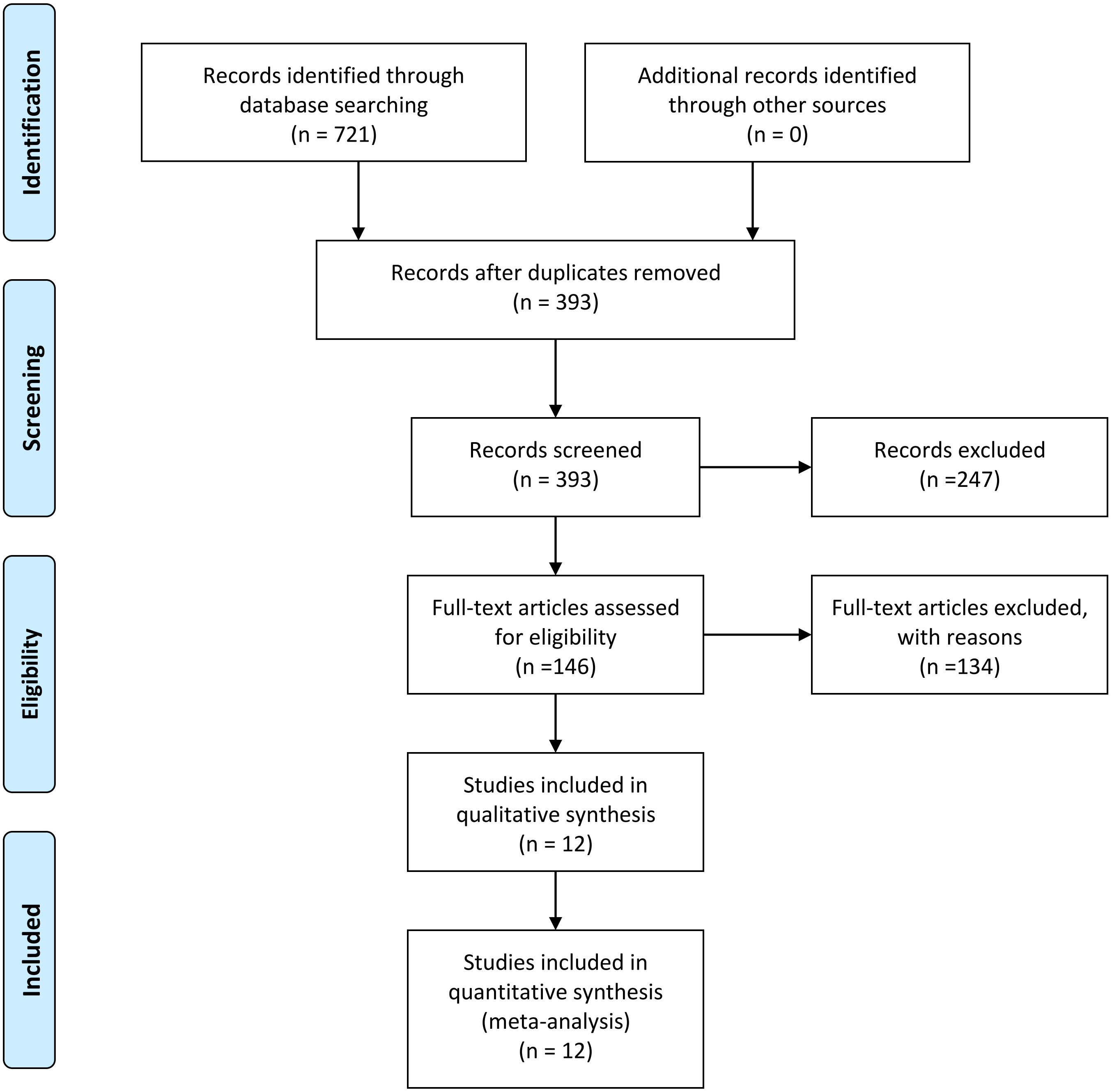

From: Moher D, Liberati A, Tetzlaff J, Altman DG, The PRISMA Group (2009). *P*referred *R*eporting *I*tems for *S*ystematic Reviews and *M*eta-*A*nalyses: The PRISMA Statement. PLoS Med 6(7): e1000097. doi:10.1371/journal.pmed1000097

For more information, visit www.prisma-statement.org.
